# Top1-PARP1 association and beyond: from DNA topology to break repair

**DOI:** 10.1093/narcan/zcab003

**Published:** 2021-02-01

**Authors:** Srijita Paul Chowdhuri, Benu Brata Das

**Affiliations:** Laboratory of Molecular Biology, School of Biological Sciences, Indian Association for the Cultivation of Science, 2A & B, Raja S. C. Mullick Road, Jadavpur, Kolkata 700032, India; Laboratory of Molecular Biology, School of Biological Sciences, Indian Association for the Cultivation of Science, 2A & B, Raja S. C. Mullick Road, Jadavpur, Kolkata 700032, India

## Abstract

Selective trapping of human topoisomerase 1 (Top1) on the DNA (Top1 cleavage complexes; Top1cc) by specific Top1-poisons triggers DNA breaks and cell death. Poly(ADP-ribose) polymerase 1 (PARP1) is an early nick sensor for trapped Top1cc. New mechanistic insights have been developed in recent years to rationalize the importance of PARP1 beyond the repair of Top1-induced DNA breaks. This review summarizes the progress in the molecular mechanisms of trapped Top1cc-induced DNA damage, PARP1 activation at DNA damage sites, PAR-dependent regulation of Top1 nuclear dynamics, and PARP1-associated molecular network for Top1cc repair. Finally, we have discussed the rationale behind the synergy between the combination of Top1 poison and PARP inhibitors in cancer chemotherapies, which is independent of the ‘PARP trapping’ phenomenon.

## INTRODUCTION

DNA repair systems provide a critical defence mechanism against exogenous DNA-damaging agents and endogenous sources that assault the stability and integrity of our genomes linked to various diseases (1–3). One of the most common forms of DNA damage that arise in cells are single-strand breaks (SSBs). The SSBs can occur from the abortive activity of DNA topoisomerase 1 (Top1), due to the covalent trapping of Top1 with the 3′-end of the DNA leading to the generation of Top1-linked DNA covalent cleavage complexes (Top1cc) (2–5). The antitumor activity of camptothecin (CPT) and other non-CPT Top1 poisons that trigger cell death through selective trapping of the Top1cc of the highly proliferating cells exploit the severity of these breaks (6). Structural alterations in the DNA, including nicks, gaps, trapped protein–DNA complexes, replication lesions and double-strand breaks (DSBs) are sensed by sensor proteins that control cell cycle checkpoints and DNA damage response (DDR) pathways (1,2,5). Poly(ADP-ribose)polymerase-1 (PARP1 or ARTD1) is a DNA nick sensor and has been proposed to play a critical role in the early detection of diverse types of DNA lesions including trapped Top1cc’s (7–10). PARP1 catalyzes the addition of ADP-ribose polymers (PAR polymers) onto itself and other chromatin proteins that modulate their biological activities during DNA repair. Remarkable advancement has been made in the past few years in the clinical application of PARP inhibitors in cancer chemotherapy particularly in tumors deficient for homologous recombination repair pathways (8,9,11,12). Some of these inhibitors impart a dominant-negative effect by trapping PARPs at DNA breaks (9,13). Concerning combination therapy, PARP inhibitors synergize with DNA base alkylating agents, cisplatin and Top1 poisons in cancers (9). This review outlines the mechanism of PARP activation, molecular networks of PARP1 for the repair of Top1-induced DNA breaks, and the rationale for the combination of PARP and Top1 poisons in cancer.

## TRAPPING OF TOPOISOMERASE 1 CLEAVAGE COMPLEXES (TOP1cc) ACCUMULATES DETRIMENTAL DNA DAMAGE

### Topoisomerase 1 (Top1)

**Figure 1.**
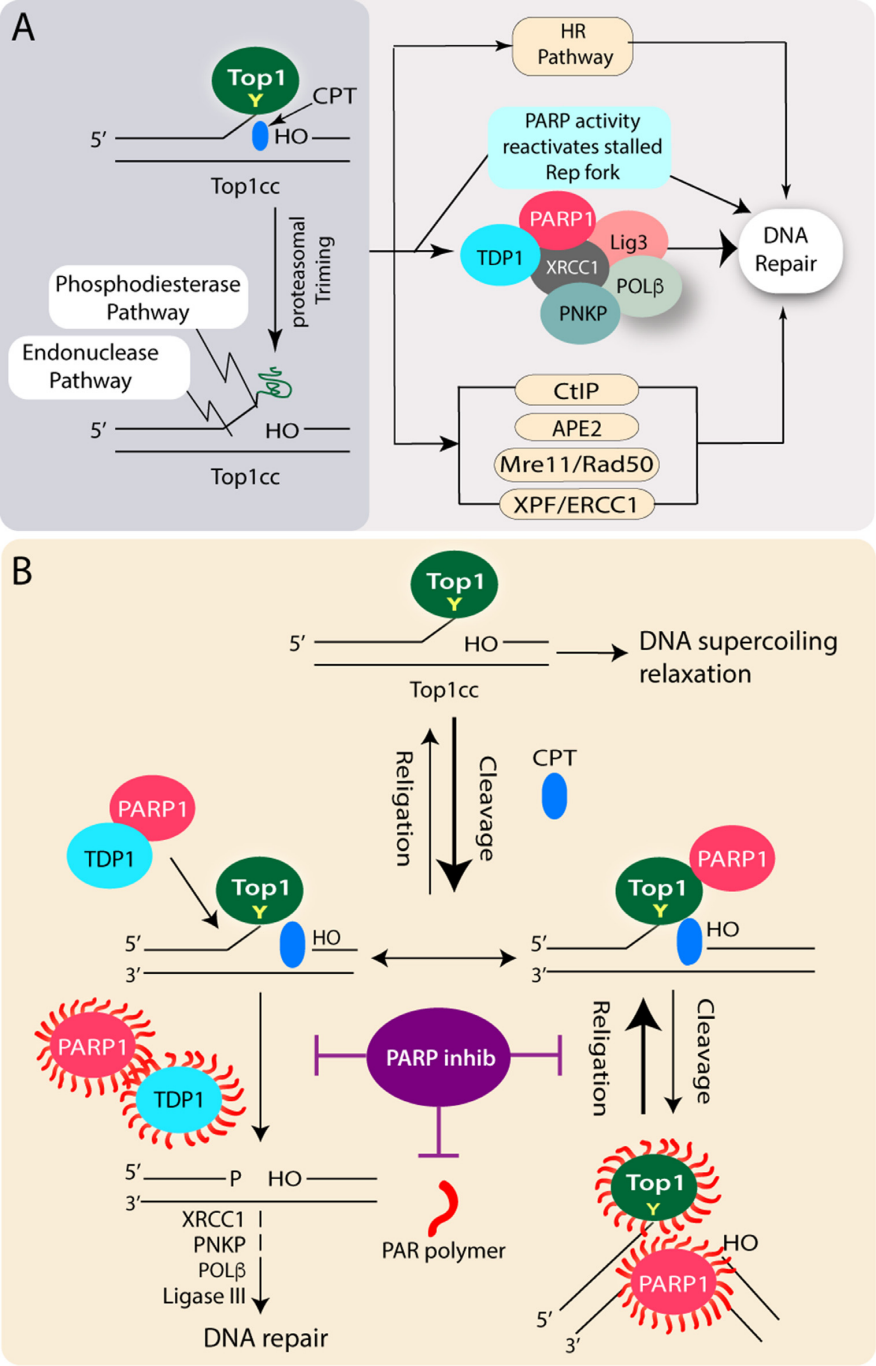
Induction of DNA damage with trapped Top1-DNA cleavage complexes (TOP1cc) and repair pathways. (**A**) Top1 cleaves one strand of duplex DNA *via* the nucleophilic attack of its active site tyrosine on the DNA phosphodiester backbone to yield a 3′-phosphotyrosyl bond. The short-lived covalent Top1-DNA cleavage complex (Top1cc) is readily trapped by Top1 poisons *i.e*. Camptothecin (CPT; blue) which binds in the interface of Top1-DNA complexes, stabilizes Top1cc, and inhibits the Top1-religation reaction. Scheme illustrating the repair pathways involved downstream to the proteasomal degradation of trapped Top1cc’s, which can be repaired in cells by broadly three pathways: (i) phosphodiesterase pathway: Excision of Top1 by TDP1 which is coupled with PARP1. PARP1 also reactivates stalled replication fork encountered by transient Top1cc; (ii) Endonuclease pathway: DNA cleavage by 3′-flap endonucleases such as XPF-ERCC1, Mre11/Rad50, CtIP and APE2; (iii) The Top1cc associated DSBs generated by replication run-off, results in a Top1-linked double-stranded end (DSE) which are repaired by homologous recombination repair. (**B**) PARP inhibitors the double-edged sword: Killing Top1 activity and inhibiting TDP1-mediated Top1cc repair. The short-lived covalent Top1-DNA cleavage complex (Top1cc) is readily reversed and facilitates DNA supercoil relaxation. The bold arrow indicates the shift in the cleavage/religation equilibrium in the presence of CPT (blue). PARylation of Top1 helps in the religation of the CPT-induced Top1 cleavage complex (Top1cc). While PARP coupling with TDP1 stimulates the excision of Top1cc by the phosphodiesterase activity of TDP1 and facilitates DNA repair. PARP inhibitors (purple) in combination with CPT abrogate Top1 and TDP1-PARylation, impair the repair of CPT-induced Top1cc, and promoting DSBs and cell death.

Human DNA Top1 (Top1) is essential as it relaxes positive DNA supercoiling in advance of replication forks and transcription complexes as well as negative supercoiling behind such complexes both in the nucleus and in the mitochondria to enable faithful transmission of our genetic information (5,14). Top1-mediated DNA supercoil relaxation involves three main steps: (i) DNA strand cleavage by a trans-esterification reaction involving a nucleophilic attack by the hydroxyl group of the active site tyrosine (Y^723^) on DNA phosphodiester bond resulting in the formation of a covalent DNA 3′-phosphotyrosyl linkage (Top1cc); (ii) DNA relaxation by controlled free rotation; and (iii) religation of the DNA strand which involves a similar trans-esterification reaction by the free DNA 5′-hydroxyl that releases the enzyme from the DNA (14). Under normal physiological conditions, the Top1 enzyme–DNA covalent complexes (Top1cc) are fleeting catalytic intermediates and normally not detectable (5). However, aborted topoisomerase catalytic activity results in the trapping of Top1 on 3′-DNA termini, which generates protein-linked DNA breaks (PDBs) (Figure 1A).

The occurrence of trapped Top1cc on DNA is markedly enhanced by the Top1 poisons, such as camptothecin (CPT) and its clinical derivatives like irinotecan and topotecan as well as several other non-CPT Top1 poisons (6,15), which bind to the interface of the ternary complex of enzyme-drug and the nicked DNA, thereby stabilizing the Top1cc and slowing the religation reaction of the nicking closing cycle (Figure 1). Top1-linked DNA single-strand break can be subsequently transformed into a DNA double-stranded break (DSB) plausibly through collision with the replication and transcription machineries (5). Trapped Top1cc’s are potent transcription-blocking DNA lesions, which may include a transient stabilization of R-loops, leading to transcription-dependent DSBs and genome instability (16,17).

Top1 has intrinsic RNA nicking activity which converts ribonucleotides embedded in cellular DNA into nicks with 2′–3′-cyclophosphate and 5′-hydroxyl ends and is responsible for the repair of misincorporated ribonucleotides during DNA replication as an alternative to RNase II activity (5). However, this activity of Top1 appears to be mutagenic and detrimental as it generates short base deletions or nicks that can then trap Top1cc (5). Therefore, repairing trapped Top1cc is an important part of DNA metabolism, which is primarily catalyzed by DNA repair proteins including Poly(ADP-ribose)Polymerase 1 and Tyrosyl-DNA Phosphodiesterase 1 (TDP1).

### Poly(ADP-ribose)polymerase-1 (PARP1)

PARP1 is a nuclear enzyme with multiple activities including the transfer of the negatively charged ADP-ribose units from nicotinamide adenine dinucleotide (NAD^+^) onto itself (a phenomenon termed as auto-PARylation) and to several histone and non-histone targets (chromatin-associated protein). PARP1 is the founding member of a superfamily of 17 enzyme isoforms that have been identified in eukaryotes and prokaryotes but not in yeast (8,18). Though PARP isoforms have different primary structures but they share homology in the domain responsible for poly(ADP-ribose) synthesis, termed PARylation (11,18). The PAR polymers are synthesized by PARP1, PARP2, PARP5A and PARP5B. The other members of the family catalyze only single ADP-ribose units and are therefore classified as mono(ADP-ribosyl)ases (MARs) (11). It is important to note that cellular PARylation is highly dynamic and fully reversible. Poly(ADP-ribose) glycohydrolase (PARG) and ADP-ribosylhydrolase 3 (ARH3) are responsible for the removal of PAR chains in almost all eukaryotic cells. The uncontrolled accumulation of PAR polymers is cytotoxic (8).

**Figure 2.**
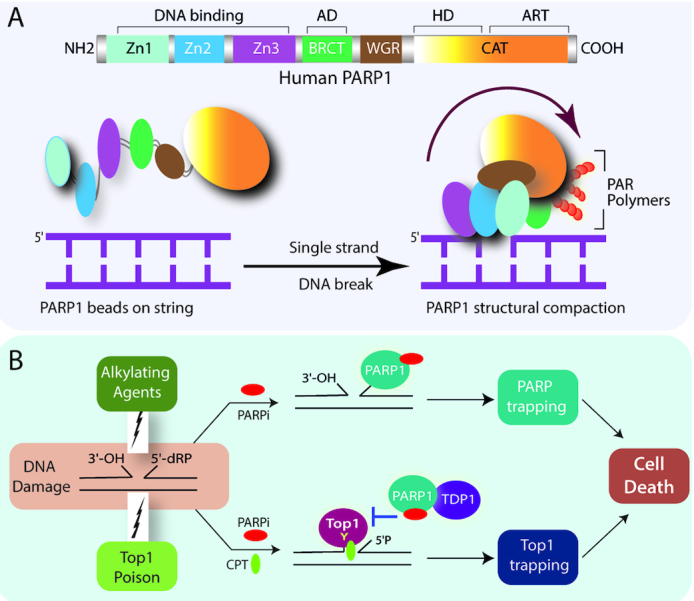
PARP1 and PARP trapping with PARP inhibitors. (**A**) PARP1 structural rearrangements facilitate the PARP1 activation at DNA damage sites. Cartoon showing the domain architecture of human PARP1 (top panel) indicating the three DNA binding zinc finger domains (Zn1, Zn2 and Zn3), BRCT (auto modification domain; AD), the WGR domain, and the Catalytic domain (CAT) composed of the helical (HD) and ART (ADP-ribosyl transferase) subdomains. PARP1 inter-domain rearrangements post DNA damage recognition (bottom panel). In absence of DNA damage PARP1 forms loose ‘beads on a string’ conformation (left) which rapidly changes into a collapsed conformation upon recruitment to DNA damage sites (right). The arrow indicates the folding of the ‘beads-on-a-string’ form of PARP1 to the compact conformation. (**B**) Top1 poisons synergize with PARP inhibitors by the catalytic inhibition of PARP1 independent of PARP trapping. The mechanistic rationale for the combination of PARP inhibitors (PARPi) with DNA damaging alkylating agents and by Top1 poisons is illustrated. The alkylating agents generate a single-nucleotide gap with 3′-OH and 5′-deoxyribose phosphate (5-dRP) groups at the ends of the breaks which remain unrepaired by PARP inhibition. Additionally, PARP gets trapped on the DNA resulting in cell death due to the competitive binding of the PARPi to the NAD+ pocket abrogating the auto-PARylation followed by the concomitant dissociation of PARP from DNA. Camptothecins (Top1 poisons; Top1i) traps Top1cc’s at the 3′-end of the DNA with 5′-sugar hydroxyl intermediates which are not preferred substrates for PARP trapping. PARP1 and TDP1 coupling involve the catalytic activity of PARP that outlines the synergy of Top1 and PARP inhibitors is through prolong Top1 trapping and cell death independent of PARP trapping.

PARP1 is composed of six domains with distinct DNA binding, catalytic and regulatory functions as shown in Figure 2A. The N-terminal region includes the DNA-binding zinc finger domains (Zn1, Zn2 and Zn3) and the central auto-modification domain harboring the BRCT (BRCA1 C-terminal) fold. The WGR (Tryptophan-Glycine-Arginine) domain is positioned next to the C-terminal catalytic domain (CAT) containing two subdomains: the helical (HD) and the ADP-ribosyltransferase (ART) (19). PARP1 stands as an exemplary to the class of multi-domain containing DDR proteins joined by a flexible *‘beads-on-a-string’* assembly by unstructured linkers that are rendered more ordered domain architecture upon binding to damaged DNA, an allosteric regulation that initiates catalytic activation and subsequent poly(ADP-ribose)-dependent DNA repair (Figure 2A) (18,19).

PARylation is one of the predominant post-translational modification (PTM) that regulates diverse biological processes such as DNA repair, oxidative stress, chromatin remodeling, regulation of transcription and apoptosis (8,11).

PARP1 is the key player for the repair of single-strand breaks (SSBR). Because SSBs are produced as an intermediate of Base-Excision Repair (BER); therefore, PARP1 is an integral part of BER and promotes DNA repair through its interaction and PARylation of several proteins involved in SSBR and BER. PARP1 has also been implicated in DNA double-strand break (DSBs) repair by regulating the upstream classical Non Homologous End Joining (cNHEJ) components (KU70, KU80, ARTEMIS and DNA-PKCs). However, the importance of PARP1 to these pathways is not completely clear (8,20). PARP1 inhibition triggers the activation of ataxia-telangiectasia-mutated kinase (ATM) (21) and is also implicated in the alternative or back up pathway for the NHEJ, which involves key factors of SSBR such as XRCC1, DNA ligase 3, and FEN1, as well as DSB repair proteins (8,11).

Further studies have suggested the role of PARP activity in protecting cells from DNA replication-associated DNA lesions. PARP1 protects stalled replication forks and promotes the restart of DNA synthesis (22). PARP activity is enriched on the DNA lagging strand of the replication fork, which is associated with PARP1 activation at unligated Okazaki fragment intermediates of DNA replication (23).

Beyond DDR pathways, PARP1 has diverse biological functions that include protein translocation, and degradation, cell division, gene silencing, RNA biology, and triggering cell death mechanism (8,18). PARylation also orchestrates a variety of processes in the nucleolar niche, which aids in the maintenance of the nucleolar structure, ribosome biogenesis, rRNA synthesis, and the epigenetic upkeep of the rDNA (8,18).

## PARP1 REGULATES SUBNUCLEAR DYNAMICS OF TOP1

Nucleoli are the subnuclear compartments without a prominent membrane, harboring repeated clusters of 200–400 ribosomal DNA (rDNA) genes. Intriguingly, the nucleolus has turned out to be the regulatory hub for multiple nuclear functions and has been attributed to stress response and DNA damage signalling. Accordingly, the dysregulation of nucleolar functions has been linked to carcinogenesis and neurological diseases (24). Due to the absence of a structural barrier between the nucleolus and the surrounding nucleoplasm, proteins can freely traffic from the nucleolus to the nucleoplasm and vice versa. However, the mechanisms by which proteins are retained in the nucleolus are still not fully understood.

Top1 is predominantly localized in the nucleolus (7,25,26). Since the nucleolus serves as the storehouse for ribosomal RNA synthesis it inevitably demands the presence of nuclear Top1 activity for the relaxation of rDNA supercoiling generated during the RNA’s replication and transcription (25).

Like Top1, PARP1 is also highly mobile in the nucleus; PARP1 is localized in the nucleoli and required for ribosomal biogenesis. Both PARP1 and PARP2 interact with nucleophosmin/B23 and accumulate in transcriptionally active nucleoli. Genetic inactivation of PARP1 activity disrupts rRNA processing and maturation (27). Top1 is an acceptor of PAR polymers which is catalyzed by PARP1 and colocalizes with PARP1 in the nucleolus and nucleoplasm throughout the cell cycle (7).

PARP1 favors a faster Top1 religation activity in the presence of CPT either through its direct interaction with Top1 or by the formation of PARylated Top1 (7,25,26). This was further supported by live-cell microscopy coupled with FRAP kinetic modeling study that shows PARP inhibitor prolongs the trapping of *in vivo* Top1cc in combination with CPT that allows accumulation of cellular DNA double-strand breaks (7,28). However, the underlying molecular mechanism by which Top1-PARylation regulates the nuclear mobility of Top1 was mostly obscure until recently, using live-cell microscopy demonstrated that disruption of PARP1 activity by PARP inhibitors delocalized Top1 from the nucleolus to the nucleoplasm which is independent of the interactions between the two proteins (7). These studies also suggest that the PARylation of Top1 serves to engage Top1 to the active sites of rDNA and rRNA synthesis.

Like PARP1, the shuttling of the repair factors including XRCC1 (X-ray repair cross-complementing protein 1), and WRN (Werner syndrome helicase), between the nucleolus and nucleoplasm is dependent on PARP1 enzymatic activity and response to DNA damage with CPT or H_2_O_2_ (29). Accordingly, the abrogation of PARP1 activity abolishes the nucleolar-nucleoplasmic shuttling of XRCC1, WRN and PARP1 (30).

## REPAIR OF TOP1cc-INDUCED DNA BREAKS

There has been a notable advancement regarding the elucidation of the repair pathways involved in the removal of trapped Top1 cleavage complexes (Top1cc). Irreversibly trapped Top1cc is trimmed through proteolysis (Figure 1A) before it is channeled to the DNA repair pathways. The Top1cc's can be repaired in cells by broadly three pathways: (i) Excision of Top1 by tyrosyl-DNA phosphodiesterase 1 (TDP1) which is coupled with PARP1, (ii) Endonuclease pathway: DNA cleavage by 3′-flap endonucleases such as XPF-ERCC1, Mre11/Rad50; CtIP and APE2, (iii) The Top1cc associated DSBs generated by replication run-off, results in a Top1-linked double-stranded end (DSE) which are repaired by homologous recombination repair (aided by BRCA1 and BRCA2 proteins), supporting the rationale behind the hypersensitivity of BRCA-deficient cancer cells to Top1 poisons (4,5,10,31) (Figure 1A).

### Tyrosyl-DNA phosphodiesterase 1 (TDP1)

The key enzyme for the excision of Top1cc is TDP1, which was discovered by Nash and colleagues (32). TDP1 is conserved in all eukaryotes and present both in the nucleus and mitochondria. TDP1 hydrolyzes the phosphodiester bond between the Top1 tyrosyl moiety and the DNA 3′-end. TDP1’s ability to resolve 3′-phosphotyrosyl linkages is consistent with its role in protecting cells against Top1-induced DNA lesions (4,10,33–35) (Figure 1A).

The ability of TDP1 to resolve 3′-phosphotyrosyl linkages is not limited to the removal of Top1-DNA adducts both in the nucleus and mitochondria but is also required for the removal of a variety of blocking lesions at the 3′-DNA ends during DNA repair which includes 3′-abasic sites and 3′-phosphoglycolate (10,35,36). TDP1 also possesses a limited DNA and RNA 3′-exonuclease activity in which a single nucleoside is removed from the 3′-hydroxyl end of the substrate (36). TDP1 is involved in the excision repair of 3′-chain-terminating anticancer and antiviral nucleosides and have weak excision activity against 5′-tyrosyl ends (10).

Human TDP1 is a neuroprotective enzyme and a homozygous mutation of TDP1 (H^493^R) is responsible for the neurodegenerative syndrome, spinocerebellar ataxia with axonal neuropathy (SCAN1) (37,38). Cells from SCAN1 patients or TDP1 knockout mice are hypersensitive to camptothecin (CPT) that selectively trap nuclear Top1-DNA covalent complexes (Top1cc) (2,3,35,39). TDP1 is critical for mitochondrial DNA (mtDNA) repair (34), accordingly SCAN1-mutant TDP1 is trapped in the specific loci of mtDNA, which increases mitochondrial DNA damage, and mitochondrial fission that leads to neuronal damage associated with SCAN1 etiology (40). Loss of TDP1 also resulted in gradual age-related cerebellar atrophy in one of the mouse model (2,35,39). Therefore, the SCAN1 pathology is a combination of both TDP1 deficiency and TDP1 trapping.

Post-translational modifications have been implicated in the recruitment, modulation of enzymatic activity, and stability of DNA damage response of TDP1. ATM (ataxia-telangiectasia mutated) and DNA-dependent protein kinase (DNA-PK) are activated in response to Top1cc-associated DSBs (1–3) that phosphorylate TDP1 at serine 81 in response to CPT or with ionizing radiation (41). CPT-induced phosphorylation at TDP1-S^81^ promotes its binding with XRCC1 and ligase IIIα, which potentially stabilizes TDP1 from degradation and enhances recruitment of pS^81^-TDP1 foci together with γH2AX and XRCC1 foci at Top1cc damage sites (41). These sites most likely correspond to the small fraction of the Top1cc’s that are converted into irreversible Top1-DNA lesions by replication and transcription (2,10,41).

Because DNA damage increases the half-life of TDP1 through phosphorylation and PARylation (28,41); therefore, the ubiquitin–proteasome system plays an important role in regulating TDP1 turnover which led to the identification of UCHL3 as the deubiquitylase enzyme controlling TDP1 proteostasis (42). TDP1-SUMOylation at lysine 111 (K111) promotes DNA repair and has been implicated in the recruitment of TDP1 at transcription-dependent Top1cc damage sites (43).

Intriguingly, protein arginine methyltransferases (PRMT5) catalyzed arginine methylation of TDP1 at residues R^361^ and R^586^ through binding with TDP1. Unlike other PTMs, arginine methylation stimulates TDP1’s-3′-phosphodiesterase activity (44). TDP1 methylation also stimulates its repair function and promotes cell survival in response to CPT and ionizing radiation.

### Coupling TDP1 and PARP1 for the repair of Top1cc-induced DNA breaks

Top1cc-induced DNA damage response is a complex signaling network that initiates with proteolysis of Top1, thereby allowing subsequent access of DNA repair enzymes including TDP1 to the Top1 active site tyrosyl-DNA linkage. Because TDP1 generates 3′-phosphate ends, its cellular activity needs to be coupled with polynucleotide kinase phosphatase (PNKP) to generate 3′-hydroxyl ends that can be extended by DNA polymerase β and DNA ligase IIIα reseals the nicks in DNA backbone (10) (Figure 1).

The involvement of PARP1 for the repair of Top1cc stems from various reports which include the hypersensitivity of PARP1 knockout cells to camptothecin (CPT); PARP inhibitors enhance the activity of CPT and its clinical derivatives both in cell cultures and in xenograft systems, and PAR accumulates in the nucleus of CPT-treated cells (5,7,10).

PARP inhibitors markedly increase DNA breaks in response to Top1cc but without a concomitant increase in Top1–DNA complexes. It is evidenced that PARP inactivation is associated with TDP1 deficiency, accordingly, PARP1 knockout cells have less TDP1 activity and the PARP inhibitor ABT-888 (veliparib) fails to sensitize Top1 poisons in TDP1-deficient cells which is implicated for the repair of Top1cc (10,28,45). Furthermore, PARP inactivation is also implicated in preventing the release of trapped Top1 from stalled replication complexes by suppressing the restart of replication forks reversed by Top1cc (8,20,23).

PARP1 appears to act as a molecular determinant for the choice between TDP1 and the endonuclease pathway for the repair of trapped Top1cc (Figure 1A). These conclusions are based on the genetic studies that suggest PARP1 and TDP1 are epistatic and show a tight coupling of TDP1 and PARP1 through direct protein–protein interaction. Furthermore, TDP1 is PARylated by PARP1 that essentially stabilizes TDP1 and enhances its recruitment to DNA damage sites without interfering with TDP1 catalytic activity. Though PARP1 facilitates XRCC1 recruitment at DNA damage sites (3), PARP1–TDP1 complexes, in turn, also recruit XRCC1 at the trapped Top1cc-DNA breaks as TDP1 deficient cells fail to generate CPT-induced XRCC1 foci (10,28).

## RATIONALE FOR THE COMBINATION OF PARP INHIBITORS WITH TOPOISOMERASE 1 POISONS

PARP inhibitors (PARPi) are a class of anticancer drugs that compete with nicotinamide adenine dinucleotide (NAD+) for the catalytically active site of PARP molecules, thereby ablating the PAR synthesizing activity of the enzyme (11). The clinical success of PARP inhibitors can be primarily reasoned to be due to the phenomenon of *synthetic lethality* wherein cells with an intrinsic perturbation or defect in one DNA repair pathway can be selectively killed or targeted by disrupting the backup pathway for the repair (11,12).

PARP inhibitors not only catalytically inactivate PARPs but also interfere with the subsequent process of release of the enzyme from the site of DNA damage; this phenomenon is called the ‘PARP trapping’ (9,13). These trapped PARPs are DNA lesions that not only block the further recruitment of other repair proteins at the DNA damage sites but also stall the DNA replication fork, and activate S-phase checkpoints leading to cell cycle arrest (11). The PARP inhibitors differ markedly in their trapping potency which corresponds to their cytotoxic potency and overall clinical doses (9,11,13).

These clinical responses for PARP inhibitors seem to be transient, as cases of revertants and PARP inhibitor resistance are on the high. Therefore, the personalized regimen of chemotherapies has started exploiting the inhibition of PARP in combination with a wide variety of DNA damaging drugs including alkylating agents (temozolomide) and Top1 poisons (camptothecin and its clinical derivatives topotecan and irinotecan). It is, however, critical to explore whether different PARP inhibitors can be considered to be equipotent in combination chemotherapies because of the PARP trapping phenomenon (11,13). Synergistic effects of PARP inhibitors combined with Top1 poisons (camptothecins and indenoisoquinolines) are well evidenced (9). The wide-scale prevalence of preclinical models exemplifying PARP inhibitors that show clinical activity in ovarian cancer, breast and prostate cancer as single agents, sensitize tumor cells to Top1 poisons *in vitro* and *in vivo* propelled combining PARP inhibitors with Top1 poison in the clinics. A combination of olaparib with topotecan or irinotecan with veliparib was effective at a much lower dose than the individual single-agents (9,46,47). It came out from one such early case of a phase I trial with Veliparib combination with topotecan or irinotecan showed significant reductions in peripheral blood mononuclear cells (PBMC) (47).

Further studies confirmed that despite the apparent similarity in the mode of action for the Top1 poisons and alkylating agents (i.e. generation of DNA SSBs which are sensed by PARP1); however, the molecular mechanisms underlying the synergy are markedly different based on the nature of the DNA breaks. The synergistic effects of PARP inhibitors in combination with TOP1 poisons is due to catalytic inhibition of PARP activity that is further supported by biochemical and cellular studies. Top1 poisons like CPT or its derivatives block the 3′-end of the DNA through Top1cc formation with a free 5′-sugar hydroxyl intermediate (9,13); however, the preferred DNA substrates for PARP1 binding are with a 5′-deoxyribose phosphate (dRP) compared to 5′-phosphate end (48,49), therefore, the specificity for the synergistic combination of PARP catalytic inhibitors with Top1 poisons is due to prolonged Top1 trapping (Figure 1B) and is independent of PARP trapping mechanism. The catalytic inhibitors of PARP abrogate PARP1–TDP1 coupling that inactivates TDP1 (28), and inhibits PAR-dependent Top1 nuclear mobility which is further trapped by Top1 poisons (7) (Figure 2B). Conversely, PARP inhibition also promotes DSB formation through replication fork collisions with CPT-induced Top1 cleavage complexes (5,8). Eventually, the failure of the synergistic cytotoxicity for a combination of Top2 inhibitors (etoposide) that trap Top2cc at DNA 5′-end with PARP inhibitors (NU1025) (11,50) does not induce PARP trapping (9), strongly suggest that PARP and Top2 inhibitors are not rational for combination therapy.

In contrast, PARP trapping and PARylation inhibition both account for the synergy with alkylating agents like methyl methanesulfonate (MMS) or Temozolomide that generates abasic sites, which are cleaved by apurinic/apyrimidinic endonuclease 1 (APE1), resulting in a single-nucleotide gap with 3′-OH and 5′- dRP at the DNA breaks that promote trapping of PARP–DNA complexes at the damage sites (Figure 2B) (9,13,48,49).

Summarily, it can be construed that when veliparib is combined with the Top1 poison (Topotecan/Irinotecan) hematologic toxicity is dose-limiting, but both agents can be administered at lower doses than the individual agent (46,47). Future studies warrant that in a more personalized tumor-specific environment the combination of PARP and Top1 poisons may benefit patients with tumors expressing high levels of PARP1 and Top1.

## CONCLUSION

PARP1 has a multifaceted role in regulating a myriad of cellular functions and a host of DNA repair pathways. PARP inhibitors as pharmacological targets in the treatment of cancers resistant to the current chemotherapeutic regimens revolutionized precision cancer therapy. However, the frequently acquired resistance to PARP inhibitors in monotherapies has spurred the need to combine PARP inhibitors with other agents. Our review summarizes that PARP1 serves as a key protein in the repair of the Top1cc’s while PARP inhibitors hold much promise as combinatorial agents when combined with Top1 poisons. The synergy that exists between Top1 poisons and PARP inhibitors seems to be driven by the catalytic inhibition of PARP1 and is not significantly incremented by the trapping potential of the PARP inhibitors (Figure 2B). Therefore, discrimination between PARP inhibitors on their ability to catalytically block the PARP1 function is necessary for future evaluation of the potential efficacy for the combination of PARP inhibitors with Top1 poison. Thus, it can be claimed that the combinatorial therapy might in a way assuage the monotherapeutic doses of the individual drugs used in tumor regression.
